# *Neisseria gonorrhoeae* antimicrobial susceptibility trends in Bangkok, Thailand, 2015–21: Enhanced Gonococcal Antimicrobial Surveillance Programme (EGASP)

**DOI:** 10.1093/jacamr/dlad139

**Published:** 2023-12-19

**Authors:** Rossaphorn Kittiyaowamarn, Natnaree Girdthep, Thitima Cherdtrakulkiat, Pongsathorn Sangprasert, Jaray Tongtoyai, Emily Weston, Andrey Borisov, Eileen F Dunne, Kittipoom Chinhiran, Joseph Woodring, Nattapon Ngarmjiratam, Silvina Masciotra, Rebekah Frankson, Pachara Sirivongrangson, Magnus Unemo, Teodora Wi

**Affiliations:** Bangrak STIs Center, Division of AIDS and STIs, Department of Disease Control and Prevention, Thailand Ministry of Public Health, Nonthaburi, Thailand; Bangrak STIs Center, Division of AIDS and STIs, Department of Disease Control and Prevention, Thailand Ministry of Public Health, Nonthaburi, Thailand; Division of HIV Prevention, U.S. Centers for Disease Control and Prevention, Atlanta, GA, USA; Division of HIV Prevention, Thailand Ministry of Public Health—U.S. Centers for Disease Control and Prevention Collaboration, Nonthaburi, Thailand; Bangrak STIs Center, Division of AIDS and STIs, Department of Disease Control and Prevention, Thailand Ministry of Public Health, Nonthaburi, Thailand; Division of HIV Prevention, U.S. Centers for Disease Control and Prevention, Atlanta, GA, USA; Division of HIV Prevention, Thailand Ministry of Public Health—U.S. Centers for Disease Control and Prevention Collaboration, Nonthaburi, Thailand; Division of STD Prevention, U.S. Centers for Disease Control and Prevention, Atlanta, GA, USA; Division of HIV Prevention, U.S. Centers for Disease Control and Prevention, Atlanta, GA, USA; Division of HIV Prevention, Thailand Ministry of Public Health—U.S. Centers for Disease Control and Prevention Collaboration, Nonthaburi, Thailand; Division of HIV Prevention, U.S. Centers for Disease Control and Prevention, Atlanta, GA, USA; Division of HIV Prevention, Thailand Ministry of Public Health—U.S. Centers for Disease Control and Prevention Collaboration, Nonthaburi, Thailand; Bangrak STIs Center, Division of AIDS and STIs, Department of Disease Control and Prevention, Thailand Ministry of Public Health, Nonthaburi, Thailand; Division of HIV Prevention, U.S. Centers for Disease Control and Prevention, Atlanta, GA, USA; Division of HIV Prevention, Thailand Ministry of Public Health—U.S. Centers for Disease Control and Prevention Collaboration, Nonthaburi, Thailand; Bangrak STIs Center, Division of AIDS and STIs, Department of Disease Control and Prevention, Thailand Ministry of Public Health, Nonthaburi, Thailand; Division of HIV Prevention, U.S. Centers for Disease Control and Prevention, Atlanta, GA, USA; Division of HIV Prevention, Thailand Ministry of Public Health—U.S. Centers for Disease Control and Prevention Collaboration, Nonthaburi, Thailand; Division of STD Prevention, U.S. Centers for Disease Control and Prevention, Atlanta, GA, USA; Department of Disease Control and Prevention, Thailand Ministry of Public Health, Nonthaburi, Thailand; WHO Collaborating Centre for Gonorrhoea and Other STIs, Department of Laboratory Medicine, Microbiology, Örebro University, Örebro, Sweden; Institute for Global Health, University College London, London, UK; Department of Global HIV, Hepatitis and STI Programmes, World Health Organization, Geneva, Switzerland

## Abstract

**Objectives:**

Rising antimicrobial resistance (AMR) in *Neisseria gonorrhoeae* is a global public health concern. Many ceftriaxone-resistant cases have been linked to Asia. In the WHO/CDC global Enhanced Gonococcal Antimicrobial Surveillance Programme (EGASP), we conducted AMR surveillance at two clinical sites in Bangkok, Thailand, 2015–21.

**Methods:**

Urethral discharge samples, from males with urethral discharge and/or dysuria, were Gram-stained and cultured. ETEST was performed to determine AMR. EGASP MIC alert values, CLSI and EUCAST breakpoints were used.

**Results:**

In 2015–21, gonococcal isolates were cultured from 1928 cases; most (64.1%) were males reporting having sex with females. The sensitivity and specificity of Gram-stained microscopy compared with culture for detection of gonococci were 97.5% and 96.6%, respectively. From 2015 to 2021, the azithromycin MIC_90_ increased from 0.125 to 1 mg/L, and the MIC_90_ of ceftriaxone and cefixime increased from 0.008 and ≤0.016 mg/L to 0.032 and 0.064 mg/L, respectively. Eight EGASP MIC alert values (in seven isolates) were identified. Five alert values were for cefixime (all resistant according to EUCAST breakpoints) and three for azithromycin (all resistant according to EUCAST breakpoints). The average annual resistance to ciprofloxacin during 2015–21 was 92%.

**Conclusions:**

A continuous high susceptibility to ceftriaxone, Thailand’s first-line gonorrhoea treatment, was found. However, the increasing MICs of ceftriaxone, cefixime and azithromycin are a substantial threat, especially considering these are the last remaining options for the treatment of gonorrhoea. To monitor AMR, continuous and quality-assured gonococcal AMR surveillance such as the Thai WHO/CDC EGASP, ideally including WGS, is imperative globally.

## Introduction

Antimicrobial-resistant gonorrhoea is a major global public health concern. The WHO estimated that there were 82 million incident cases of gonorrhoea worldwide in 2020.^1^ The prevalence of *Neisseria gonorrhoeae* is highest in low- and middle-income countries in Southeast Asia and the Western Pacific.^1^ In Thailand, the rates of bacterial sexually transmitted infections (STIs) including gonorrhoea, non-gonococcal urethritis, syphilis, chancroid and lymphogranuloma venereum have increased in recent years, from 28.8 per 100 000 population in 2017 to 33.6 per 100 000 population in 2020. Gonorrhoea was the most common bacterial STI in Thailand until 2019, but the number of cases has decreased thereafter.^2^ With limited resources, especially in rural areas of Thailand, syndromic management for urethral discharge and urethritis is frequently used and aetiological diagnosis is rare. Consequently, the true national incidence of gonorrhoea and gonococcal AMR remains largely unknown.

WHO ranked *N. gonorrhoeae* as a high-priority pathogen for AMR in 2017.^3,4^ The emergence of MDR and XDR *N. gonorrhoeae* threatens the use of the last remaining recommended effective class of antibiotics, the extended-spectrum third-generation cephalosporins.^5–12^ Gonococcal infections acquired in or from Asia account for most of the confirmed ceftriaxone treatment failures, with several ceftriaxone-resistant strains that emerged in Asia spreading globally.^13–24^ In 2016, the first documented gonorrhoea treatment failure with the recommended dual therapy of ceftriaxone plus azithromycin was reported from a patient in the UK who had been infected in Japan.^19^ Furthermore, the first two XDR gonococcal strains with ceftriaxone resistance combined with high-level azithromycin resistance were cultured in the UK^20^ and Australia^21^ in 2018 (indistinguishable strain), and in Austria in 2022,^18^ respectively. The case in the UK in 2018 was infected in Thailand, one of the two isolates in Australia was also linked to South-East Asia, and the different Austrian strain was acquired in Cambodia.^18^ Notably, a gonococcal isolate recently detected in France was identical to the XDR strain from Austria, and also this strain had links to Cambodia.^22^ Additionally, the internationally spreading ceftriaxone-resistant FC428 strain, first reported in Japan in 2015,^23^ including its evolving subtypes, has been reported in many countries.^24^ Finally, in 2017–18 the WHO Global Antimicrobial Surveillance Programme (GASP) reported decreased susceptibility or resistance to ceftriaxone, cefixime and azithromycin in 31%, 47% and 84% of reporting countries, respectively, and to ciprofloxacin in all (100%) of the 70 reporting countries.^16^ However, continuous and quality-assured data on *N. gonorrhoeae* antimicrobial resistance (AMR) in Asia remain sparse as surveillance systems to monitor *N. gonorrhoeae* AMR are insufficient or absent in many Asian countries and globally overall.^16^

The WHO Global Action Plan on AMR calls for significant improvements to strengthen AMR surveillance and research.^25^  *N. gonorrhoeae* AMR surveillance aims to detect and monitor AMR trends and the distribution of AMR in subgroups [e.g. MSM, transgender women (TGW) and sex workers], and to identify novel emerging AMR in order to inform the prevention and containment of AMR by identifying early signals that necessitate changes in recommended antibiotic treatment strategies.^25,26^ Accordingly, *N. gonorrhoeae* AMR surveillance activities provide essential data to inform revisions of evidence-based treatment recommendations as well as antibiotic stewardship. The global Enhanced Gonococcal Antimicrobial Surveillance Programme (EGASP), a partnership between the WHO and US CDC, is a prime example of how the surveillance of AMR in *N. gonorrhoeae* can be strengthened, particularly in countries with a high burden of gonococcal infections.^27,28^

The WHO/CDC EGASP was initiated in 2015, and Thailand was the first country to implement programme activities as a collaboration between the Thailand Ministry of Public Health (MOPH), the WHO and the US CDC. EGASP was established to provide enhanced surveillance of AMR in *N. gonorrhoeae* isolates using standardized laboratory and quality assurance procedures and to collect the epidemiological characteristics of persons with infection. The EGASP has subsequently expanded to more countries globally with other disease surveillance programmes learning from Thai leaders about the successes and challenges in establishing and maintaining EGASP. In the first year of EGASP in Bangkok, Thailand from November 2015 to October 2016, the gonococcal isolates (*n* = 590) were all susceptible to extended-spectrum cephalosporins (ESCs, ceftriaxone and cefixime) and azithromycin, while resistance to ciprofloxacin was high (92.4%).^29^

In the present manuscript, we report and analyse the EGASP results in Bangkok, Thailand from November 2015 to December 2021, elucidating AMR trends in *N. gonorrhoeae* over a 6 year period, and highlighting the worrying decrease in antibiotic susceptibility in recent years.

## Materials and methods

### Patients and collection of demographic, clinical and behavioural data

From November 2015 to December 2021, demographic, clinical and behavioural data were obtained from males presenting with urethral discharge and/or dysuria at two clinical sites in Bangkok, namely the Bangrak STIs Center (BSC) and the US CDC’s Silom Community Clinic (SCC) located at the Hospital of Tropical Medicine. Urethral discharge specimens were also collected for Gram-stained microscopy and culture. The BSC provides STI screening and treatment to the general population, including MSM, TGW and sex workers. The SCC is a research clinic that focuses on providing HIV and STI screening and prevention services to MSM and TGW. At the BSC, all individual males presenting with urethral discharge and/or dysuria were included in the EGASP surveillance; however, SCC only included data from males with urethral discharge and/or dysuria that were not participating in concurrent clinical research studies related to *N. gonorrhoeae* diagnosis and treatment. If males previously diagnosed with gonococcal infection were infected again, they were reported as a repeat gonorrhoea case.

As part of healthcare service provision, demographic (e.g. age), clinical (e.g. symptoms and signs, previous antibiotic use, treatment received) and behavioural data (e.g. sexual behaviours) were collected from persons presenting with urethral discharge and/or dysuria using paper-based and electronic questionnaires. The EGASP surveillance data were de-identified, with a unique surveillance identification (ID) number assigned to each EGASP surveillance record and no personally identifiable information (PII) was transferred to the EGASP database.

All patients were treated according to the Thailand MOPH STI guidelines^30,31^ on their first visit, based on clinical evaluations and microscopy results. Consequently, if Gram staining showed Gram-negative intracellular diplococci and non-gonococcal urethritis had not been excluded, patients received ceftriaxone 250 mg (from 2015 to April 2019) and 500 mg (from May 2019) plus azithromycin/doxycycline. Patients who only had polymorphonuclear cells ≥5 cells/oil immersion field were treated as non-gonococcal urethritis cases.^32^

The EGASP has undergone reviews by the Thailand MOPH, WHO and US CDC and it has been classified as a routine public health surveillance activity, i.e. not human-subject research. EGASP is carried out as a part of routine healthcare practice and no PII is collected or stored. Accordingly, no separate ethical approval is required.

### Sampling of urethral specimens and diagnosis of gonorrhoea

Trained nurses collected urethral discharge using sterile aluminium loops or Dacron swabs. Specimens were inoculated directly on to selective culture agar medium (modified Thayer–Martin medium, which contains nystatin, colistin, vancomycin and trimethoprim for selectiveness). Another swab was used to collect urethral specimens for Gram staining. Inoculated agar plates were incubated, as soon as possible but within 30 min in each STI reference laboratory, at 36°C ± 1°C in a 5% CO_2_-enriched humid atmosphere for up to 48 h (examined daily). *N. gonorrhoeae* identification was performed using colony characteristics on selective culture agar media, Gram-stained microscopy, oxidase test, superoxol test and a rapid micro carbohydrate test.^32^

### Antimicrobial susceptibility testing

MICs (mg/L) of ceftriaxone, cefixime, azithromycin, gentamicin and ciprofloxacin were determined using the ETEST (bioMérieux, Marcy-l’Étoile, France), in accordance with the manufacturer’s instructions. *N. gonorrhoeae* ATCC 49226 and 2016 WHO reference strains (WHO L, WHO M and WHO U)^33^ were included in each batch of testing for internal quality control purposes. External quality assessments were provided annually by the Division of STD Prevention (DSTDP), US CDC.

The following EGASP MIC alert values were used: ceftriaxone MIC ≥ 0.125 mg/L, cefixime MIC ≥ 0.25 mg/L, azithromycin MIC ≥ 2 mg/L and gentamicin MIC ≥ 32 mg/L.^28^ All EGASP MIC alert values were verified by repeated testing. No ciprofloxacin EGASP MIC alert value was used because the resistance to ciprofloxacin was over 90% in our setting. The EUCAST 2023 breakpoints^34^ were also used: ceftriaxone resistance MIC > 0.125 mg/L; cefixime resistance MIC > 0.125 mg/L; azithromycin resistance MIC > 1 mg/L (based on the epidemiological cut-off); and ciprofloxacin resistance MIC > 0.064 mg/L. Finally, in accordance with the Thai EGASP protocol,^29^ the CLSI 2023 breakpoints were used; non-susceptibility was defined as MIC > 0.25 mg/L for ceftriaxone and cefixime, and MIC > 1 mg/L for azithromycin, and resistance to ciprofloxacin was defined as MIC ≥ 1 mg/L.^35^ No clinical breakpoints for gentamicin have been established by CLSI or EUCAST (https://www.eucast.org/clinical_breakpoints).^34,35^

### Data analysis

Descriptive statistics (counts, proportions etc.) were used to characterize the demographic, behavioural and clinical attributes of participants in EGASP (cases), as well as instances of recurrent gonococcal infections and documented prior treatments. The sensitivity and specificity of Gram staining for presumptive identification of *N. gonorrhoeae* were evaluated in comparison with culture. We performed a multivariable analysis to identify factors associated with repeat *N. gonorrhoeae* infections. ORs and 95% CIs were calculated. Descriptive statistics were performed for all *N. gonorrhoeae* isolates with available antimicrobial susceptibility data, both annually and cumulatively. MIC_50_, MIC_90_ and MIC ranges were determined. The significance level was set at *P* < 0.05. Data analysis was conducted using Stata Version 12.0 (StataCorp, College Station, TX, USA).

## Results

### EGASP male participants, 2015–21

From 2015 to 2021, a total of 3848 urethral specimens were collected for inclusion in EGASP from males with urethritis who sought testing at BSC and SCC. A total of 1678 males (who contributed 1928 specimens) were diagnosed with *N. gonorrhoeae* infection and of those, 1098 (65.4%) reported sex with females only. Among EGASP participants from BSC, 21.9% (298/1360) were MSM while 86.8% (276/318) of EGASP participants from SCC were MSM. The median reported number of males per year was 512 (range: 150–1013). For *N. gonorrhoeae* culture-positive cases, the median age at the time of diagnosis was 29 years (range: 12–81 years); *N. gonorrhoeae* culture positivity was higher among participants aged ≤45 years compared with those aged >45 years (Table 1).

**Table 1.** Bivariable and multivariable analysis of epidemiological factors associated with gonococcal infections among males with urethritis, EGASP, Bangkok, Thailand, November 2015–December 2021

Characteristic	All NG patients		Single NG infection		Repeat NG infection					
	*N*	%	*N*	%	*N*	%	OR	95% CI	Adjusted OR^a^	95% CI
Total	1678	100	1519	100	159	100				
Clinic										
BSC	1360	81.0	1249	82.2	111	69.8	ref		ref	
SCC	318	19.0	270	17.8	48	30.2	2.0	1.39–2.88	1.7	1.11–2.74
Age (years) at first positive culture										
<25	512	30.5	465	30.6	47	29.6	ref		ref	
25–45	909	54.2	823	54.2	86	54.1	1.0	0.72–1.51	1.1	0.73–1.56
>45	257	15.3	231	15.2	26	16.4	1.1	0.68–1.84	1.4	0.82–2.38
Sex of sexual partner(s)										
Male only	493	29.4	430	28.3	63	39.6	1.7	1.22–2.43	1.4	0.91–2.16
Female and male	81	4.8	71	4.7	10	6.3	1.7	0.82–3.33	1.5	0.71–3.03
Female only	1098	65.4	1012	66.7	86	54.1	ref		ref	
Unreported	6	0.3	5	0.3	0	0	N/A		N/A	
Antibiotic treatment in previous 14 days										
Yes	451	26.9	409	26.9	42	26.4	1.0	0.67–1.41	1.1	0.74–1.59
No	1206	71.9	1091	71.8	115	72.3	ref		ref	
Unknown	21	1.3	19	1.3	2	1.26	N/A		N/A	

NG, *Neisseria gonorrhoeae*; N/A, not applicable (not included in calculations because the number was too low).

Exposure to antibiotics in the past 2 weeks was reported in 26.9% of diagnosed cases (Table 1). The majority (99.7%) of gonorrhoea cases (either diagnosed with Gram stain or culture) were treated with antibiotics recommended by the Thailand MOPH national treatment guidelines,^30,31^ and 97% of gonorrhoea cases were treated with ceftriaxone with the recommended dose. Other treatments were prescribed to patients who concurrently participated in EGASP and research studies evaluating new STI agents at BSC (i.e. zoliflodacin) or patients with a drug allergy history to penicillin.

A total of 159 (9.5%) males with initial gonococcal infection experienced ≥1 repeat gonococcal infection(s); this varied by clinical site: 15.1% (48/318) at SCC and 8.2% (111/1360) at BSC (Table 1). The proportion of males with a history of two repeated gonorrhoea episodes was 6.7% (112/1678), and the proportion of males with a history of more than two repeat gonococcal infections was 2.8% (47/1678). Multivariable analysis of factors independently associated with repeat *N. gonorrhoeae* infection showed that undergoing testing at SCC was the only factor associated with repeat gonococcal infection (OR 1.7; 95% CI 1.1–2.7) (Table 1).

### Detection of N. gonorrhoeae

Of the total 3848 urethral specimens collected for inclusion in EGASP, *N. gonorrhoeae* infection was confirmed by culture in 1928 specimens (50.1%). Of these, 1519 (78.8%) specimens were obtained from newly infected males and 409 (21.2%) specimens from males who had ≥1 repeat *N. gonorrhoeae* infection. The percentages of Gram-stained microscopy-positive samples were similar to the percentages of *N. gonorrhoeae* culture-positive samples (Table 2). The sensitivity and specificity of Gram-stained microscopy to presumptively identify *N. gonorrhoeae* compared with culture of *N. gonorrhoeae* were 97.5% (95% CI 96.8–98.1) and 96.6% (95% CI 95.9–97.2), respectively. The number of *N. gonorrhoeae* culture-positive samples each year from 2015 to 2021 was 78 (November 2015 to December 2015), 613, 376, 358, 250, 143 and 110, respectively.

**Table 2.** Characteristics of collected urethral specimens from males with urethritis, EGASP, Bangkok, Thailand, November 2015–December 2021

Characteristics	*N*	%
Total	3848	100
Clinic		
BSC	3013	78.3
SCC	835	21.7
Gram stain positive at first positive visit	1593	41.4
Gram stain positive (including repeat infection)	1946	50.6
NG culture positive^a^	1928	50.1
Number with AST results^b^	1927	50.1

AST, antimicrobial susceptibility testing.

^a^One case was missing culture identification results.

^b^One case was missing AST results.

### Antimicrobial susceptibility testing

**Figure 1.**
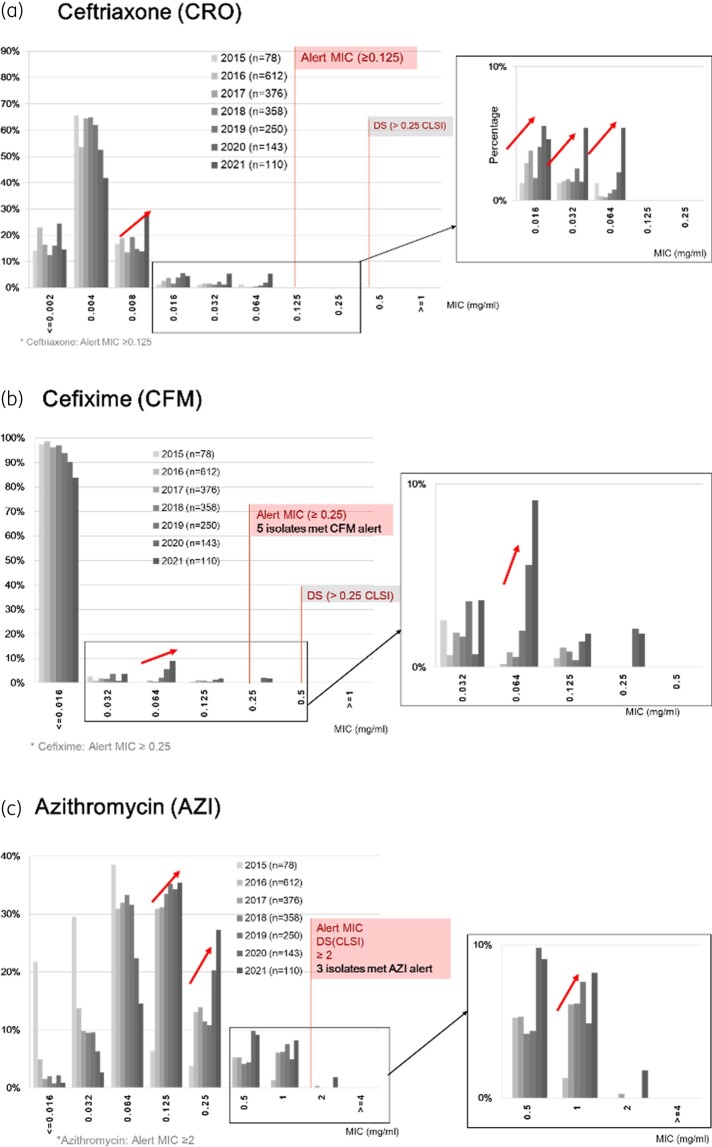
Distribution of MIC values of *N. gonorrhoeae* isolates, EGASP, Bangkok, Thailand, November 2015–December 2021. (a) Ceftriaxone (CRO), (b) cefixime (CFM), (c) azithromycin (AZM). EGASP MIC alert values are included in red boxes.^28^ The red arrows highlight the marked increases in the proportion of isolates with higher MICs in each year. DS, decreased susceptibility.

The MIC_50_, MIC_90_, and minimum and maximum MIC values for all antimicrobials and isolates by year are shown in Table 3. All *N. gonorrhoeae* isolates were susceptible to ceftriaxone. Ceftriaxone MICs ranged from ≤0.002 to 0.064 mg/L (Table 3) and over the years there was an increase in the proportion of isolates with ceftriaxone MICs of 0.064 mg/L (Figure 1a). The MICs of cefixime varied from ≤0.016 to 0.25 mg/L (Table 3, Figure 1b). MIC_50_ and MIC_90_ values for ceftriaxone and cefixime were stable from 2015 to 2020, but the MIC_90_ values for both antibiotics were significantly higher in 2021 (Fisher’s exact *P* value < 0.001). Accordingly, the MIC_90_ of ceftriaxone increased from 0.008 in 2020 to 0.032 mg/L in 2021 and the MIC_90_ for cefixime increased from ≤0.016 in 2020 to 0.064 mg/L in 2021. The azithromycin MIC_90_ increased from 0.125 in 2015 to 1 mg/L in 2021 (Table 3, Figure 1c). The percentage of ciprofloxacin-resistant isolates was 92.1% (range: 89.6%–96.4%) (Table 3).

**Table 3.** MICs (mg/L) of *N. gonorrhoeae* isolates collected from males with urethritis, EGASP, Bangkok, Thailand, November 2015–December 2021

Antibiotic	MIC values (mg/L)	2015 ^a^ (*n* = 78)	2016 (*n* = 613)	2017 (*n* = 376)	2018 (*n* = 358)	2019 (*n* = 250)	2020 (*n* = 143)	2021 (*n* = 110)
Ceftriaxone	Min MIC	≤0.002	≤0.002	≤0.002	≤0.002	≤0.002	≤0.002	≤0.002
	MIC_50_	0.004	0.004	0.004	0.004	0.004	0.004	0.004
	MIC_90_	0.008	0.008	0.008	0.008	0.008	0.008	0.032
	Max MIC	0.064	0.064	0.064	0.064	0.064	0.064	0.064
	% with MIC ≥0.125 (EGASP alert value)	0	0	0	0	0	0	0
	% with MIC >0.125 (EUCAST, resistant)	0	0	0	0	0	0	0
Cefixime	Min MIC	≤0.016	≤0.016	≤0.016	≤0.016	≤0.016	≤0.016	≤0.016
	MIC_50_	≤0.016	≤0.016	≤0.016	≤0.016	≤0.016	≤0.016	≤0.016
	MIC_90_	≤0.016	≤0.016	≤0.016	≤0.016	≤0.016	≤0.016	0.064
	Max MIC	0.032	0.125	0.125	0.125	0.125	0.25	0.25
	% with MIC >0.25 (EGASP alert value; EUCAST, resistant)	0	0	0	0	0	2.1 (*n* = 3)	1.8 (*n* = 2)
Azithromycin	Min MIC	≤0.016	≤0.016	≤0.016	≤0.016	≤0.016	≤0.016	≤0.016
	MIC_50_	0.032	0.125	0.125	0.125	0.125	0.125	0.125
	MIC_90_	0.125	0.25	0.5	0.5	0.5	0.5	1
	Max MIC	0.25	1	2	1	1	1	2
	% with MIC ≥2 (EGASP alert value; EUCAST, resistant)	0	0	0.3 (*n* = 1)	0	0	0	1.8 (*n* = 2)
Gentamicin	Min MIC	2	1	1	2	2	4	2
	MIC_50_	4	4	4	4	4	8	8
	MIC_90_	8	8	8	8	8	8	8
	Max MIC	8	8	8	8	8	16	8
	% with MIC = 16 (EGASP alert value)	0	0	0	0	0	0.7 (*n* = 1)	0
Ciprofloxacin	Min MIC	1	≤0.002	≤0.002	≤0.002	≤0.002	≤0.002	≤0.002
	MIC_50_	2	2	2	2	2	2	2
	MIC_90_	16	8	8	8	8	8	8
	Max MIC	≥32	≥32	≥32	≥32	≥32	≥32	≥32
	% with MIC ≥ 1 (CLSI, resistant)	100.0 (*n* = 78)	91.5 (*n* = 560)	92.0 (*n* = 346)	90.5 (*n* = 324)	89.6 (*n* = 224)	92.3 (*n* = 132)	96.4 (*n* = 106)

EGASP MIC alert values; ceftriaxone MIC ≥ 0.125 mg/L, cefixime MIC ≥ 0.25 mg/L, azithromycin MIC ≥ 2 mg/L and gentamicin MIC ≥ 32 mg/L.^28^

The CLSI guidelines released in 2021 define non-susceptibility to ESCs (i.e. ceftriaxone and cefixime) as MIC > 0.25 mg/L and azithromycin as MIC > 1 mg/L. Ciprofloxacin resistance is defined as MIC ≥ 1 mg/L.^35^

The EUCAST defines ceftriaxone and cefixime resistance as MIC > 0.125 mg/L, azithromycin resistance as MIC > 1 mg/L (based on epidemiological cut-off) and ciprofloxacin resistance as MIC > 0.06 mg/L.^34^

^a^November and December only.

There were seven isolates with verified EGASP MIC alert values (*n* = 8), as shown in Table 4. No isolates with an EGASP MIC alert value for ceftriaxone were found. However, five isolates with cefixime EGASP MIC alert values (5/1927; 0.26%), four from SCC and one from BSC, were verified in 2020 (*n* = 3; 2.1%) and 2021 (*n* = 2; 1.8%). All of these isolates had a cefixime MIC of 0.25 mg/L, i.e. the isolates were resistant to cefixime according to the EUCAST breakpoints^34^ and exactly at the breakpoint for non-susceptibility to cefixime according to the CLSI breakpoints.^35^ The ceftriaxone MICs of these cefixime MIC alert isolates were 0.032–0.064 mg/L. The first isolate with an EGASP MIC alert value for azithromycin was verified in 2017 and two additional isolates were found in 2021. Consequently, 0.16% (3/1927) of all isolates (1.8% in 2021) were resistant to azithromycin according to the EUCAST breakpoints^34^ and non-susceptible to azithromycin according to the CLSI breakpoints.^35^ One isolate showed EGASP MIC alert values for both cefixime (MIC = 0.25 mg/L) and azithromycin (MIC = 2 mg/L). All seven alert cases (eight MIC alert values) were identified at the time of first gonorrhoea diagnosis in this surveillance, with all participants stating that they had not received any antibiotic in the last 14 days before coming to either clinic.

**Table 4.** Characteristics of *N. gonorrhoeae* isolates collected from males with urethritis with MIC alert values (bold letters), EGASP,^28^ Bangkok, Thailand, November 2015–December 2021

Case	Year detected	Clinic name	Age	Sex of sexual partner	Number of treatments received	Previous antibiotic used	MIC values (mg/L; ETEST)				
							CRO	CFM	AZM	GEN	CIP
1	2017	BSC	19	Male	1^st^	No	0.004	0.016	**2^a^**	8	8
2	2020	BSC	29	Male	1^st^	No	0.064	**0**.**25^b^**	0.5	8	32
3	2020	SCC	35	Male	1^st^	No	0.032	**0**.**25^b^**	1	8	4
4	2020	SCC	27	Male	1^st^	No	0.032	**0**.**25^b^**	1	8	8
5	2021	SCC	27	Female	1^st^	No	0.004	0.016	**2^a^**	8	8
6	2021	SCC	43	Male	1^st^	No	0.032	**0**.**25^b^**	**2^a^**	8	8
7	2021	SCC	24	Male	1^st^	No	0.064	**0**.**25^b^**	1	8	8

CRO, ceftriaxone; CFM, cefixime; AZM, azithromycin; GEN, gentamicin; CIP, ciprofloxacin.

^a^Resistant to azithromycin according to the current EUCAST breakpoints.^34^

^b^Resistant to cefixime according to the current EUCAST breakpoints.^34^

## Discussion

Gonorrhoea remains a major public health concern globally, and antimicrobial-resistant *N. gonorrhoeae* is becoming more prevalent internationally. In 2017, the WHO published a list of 12 priority pathogens that pose the greatest threat to human public health due to AMR, with *N. gonorrhoeae* included in the second highest category of urgency.^3^ Therefore, having quality-assured surveillance systems to monitor AMR *N. gonorrhoeae* remains a global priority. For the management of symptomatic urethritis, the WHO recommends treating based on diagnostic test results, where feasible.^36^ This recommendation is supported by our surveillance data, which show that nearly 50% of males who had urethral discharge and/or dysuria did not have gonorrhoea. Although culture has good sensitivity and specificity in detecting urethral gonorrhoea in males, it takes at least 2–3 days to get the results and typically requires a patient to return to the clinic for treatment. Our data highlight that microscopy of Gram-stained urethral smears in symptomatic male urethritis patients can be effectively used for presumptive diagnosis of gonorrhoea and empirical treatment; the sensitivity and specificity of microscopy of Gram-stained smears in our study was high when compared with culture, which aligns with previous studies.^37^

From the 3848 urethral specimens included during 2015–21, *N. gonorrhoeae* was cultured from 50% of specimens; in comparison, South Africa has reported *N. gonorrhoeae* culture positivity of 70% in male urethritis cases.^38^ The lower prevalence of *N. gonorrhoeae* culture positivity in Thai cases of urethritis may reflect other causes of urethritis, such as *Chlamydia trachomatis* or *Mycoplasma genitalium*, but also prior antimicrobial use, which was high in the present study (26.4%). The high rate of previous antibiotic use among our participants exemplifies current challenges with antibiotic stewardship in Thailand, where a person can largely get most oral medications from most pharmacies without the need for a prescription from a healthcare provider.^39^ The most updated STI treatment guidelines in Thailand from 2019 recommends ceftriaxone 500 mg as monotherapy for gonorrhoea treatment; however, if non-gonococcal urethritis cannot be excluded on the same day, azithromycin or doxycycline should also be given.^31^ Because of the limited use of rapid molecular diagnosis in Thailand, this dual therapy has been frequently used for syndromic treatment.

Since 1988, ceftriaxone has been the recommended first-line treatment for gonorrhoea in Thailand, and azithromycin has been used to treat non-gonococcal urethritis.^40^ As shown in the present paper, the vast majority (99.7%; 1923/1928) of isolates remained susceptible to both ceftriaxone and cefixime during 2015–21. Although a lower number of collected isolates in 2020 and 2021 may have been the result of social and physical restrictions due to the COVID-19 pandemic and the reluctance of patients to visit healthcare clinics for care, the MIC_90_ for both these ESCs had significantly increased in 2021. Furthermore, three (2.1%) and two (1.8%) isolates in 2020 and 2021, respectively, displayed EGASP MIC alert values for cefixime (MIC = 0.25 mg/L) and were resistant to cefixime using the EUCAST breakpoints.^34^ Prior to 2020, there were no EGASP MIC alerts for cefixime, i.e. no cefixime-resistant isolates detected in the EGASP Thailand programme. A cefixime MIC of 0.25 mg/L has not been previously reported in any *N. gonorrhoeae* isolate cultured in Thailand. A prior study at BSC found no isolates with elevated ceftriaxone or cefixime MIC among 99 examined gonococcal isolates in 2018.^41^

Due to the increasing number of isolates with decreased susceptibility to cefixime since 2015, cefixime was discontinued as the recommended first-line treatment of gonorrhoea in 2019 in Thailand. Despite this, no isolate with EGASP MIC alert values or resistance/non-susceptibility to ceftriaxone has been detected in Thailand to date; however, increased awareness of potential ceftriaxone resistance remains crucial to national authorities. The first report of decreased susceptibility to ceftriaxone (MIC = 0.125 mg/L) in Thailand was in 2017, in isolates from urethral gonorrhoea in long-term resident males from South Africa and Australia.^42^ Those cases were successfully treated with ceftriaxone 250 mg plus azithromycin 1 g. Worryingly, both males were lost to follow-up and no partners received treatment. Furthermore, in 2018, a case reported in the UK with travel links to Thailand showed combined ceftriaxone resistance (MIC = 0.5 mg/L) and high-level azithromycin resistance (MIC > 256 mg/L), which failed treatment with ceftriaxone 1 g but was finally cured with 3 days of ertapenem 1 g daily.^20^

Azithromycin is frequently used for non-gonococcal urethritis treatment and co-treatment of gonorrhoea; however, only 0.3% of isolates in 2017 and 1.8% of isolates in 2021 had verified EGASP MIC alert values for azithromycin. These isolates were resistant to azithromycin according to the EUCAST breakpoints.^34^ A previous study from 2018 also described 2% resistance to azithromycin among gonococcal isolates from BSC.^41^ This is a substantially lower azithromycin resistance level compared with what has been reported from many other countries in the WHO South East Asian Region (SEAR) and Western Pacific Region (WPR).^16,43^ Continuous close monitoring of azithromycin susceptibility is essential as the MIC_90_ of azithromycin substantially increased from 2015 to 2021, and 1.8% of isolates in 2021 were resistant to azithromycin according to the EUCAST guidelines.^34^ In the present study, we did not observe any association between either previous antibiotic use or repeat infection with EGASP MIC alert values for any antimicrobial.

Ciprofloxacin resistance remained very high during 2015–21, ranging from 89.6% to 96.4% per year, which is similar to the majority of countries in the WHO SEAR and WPR.^16,43^ In Thailand, fluoroquinolones were discontinued as a recommended gonorrhoea treatment in 2004.^44^

Around 30% of our EGASP cases were MSM. Interestingly, all five EGASP MIC alert isolates for cefixime (cefixime resistant according to EUCAST breakpoints)^34^ were cultured from MSM. Repeated screening recommendation for gonorrhoea in MSM with infection every 3–6 months may be beneficial. Enhanced surveillance of gonococcal AMR in specific populations including MSM should be considered globally. A test of cure should be performed when the gonococcal isolate has an EGASP MIC alert value for any antimicrobial used for treatment and, additionally, sexual partner(s) should ideally be screened.

There are limitations to EGASP surveillance in Thailand, including that only symptomatic males with urethral discharge or urethritis, and no females or cases with extra-genital infections, such as oropharyngeal or rectal, were included. Accordingly, our data are not representative of females, key populations (e.g. MSM), settings beyond Bangkok, or extra-genital gonococcal infections. Furthermore, predominantly heterosexual males were included; larger proportions of MSM, TGW and sex workers would be valuable to include in future surveillance. Currently, work is in progress to enhance and expand EGASP in Thailand, for several reasons, including to improve the representativeness, include oropharyngeal and rectal samples, add an additional site outside Bangkok (with an increased number of sex workers), and implement WGS of the gonococcal isolates.

In conclusion, this is a contemporary surveillance report of gonococcal AMR from EGASP in Bangkok, and the most comprehensive longitudinal data to date from Thailand. Although no gonococcal isolates with ceftriaxone resistance according to current EUCAST breakpoints^34^ or non-susceptibility based on current CLSI breakpoints^35^ were detected, the increasing MIC_90_s of ceftriaxone, cefixime and azithromycin and isolates with resistance to cefixime and azithromycin (according to EUCAST breakpoints)^34^ are major concerns for the potential of emerging ceftriaxone-resistant and untreatable gonorrhoea as other treatment options are absent in Thailand and worldwide. Similar to our Thai EGASP, enhanced, continuous and quality-assured *N. gonorrhoeae* AMR surveillance, ideally including WGS, in many additional Asian countries could be useful.
